# Synergistic Effects of High-Intensity Interval Training and *Asparagus officinalis* L. Root Extract Supplementation on Metabolic Regulation, Oxidative Stress, and Inflammation in Overweight and Obese Adults

**DOI:** 10.3390/ijms262412054

**Published:** 2025-12-15

**Authors:** Piyapong Prasertsri, Tadsawiya Padkao, Orachorn Boonla, Surachat Buddhisa, Nattaphol Prakobkaew, Siriporn Sripinyowanich, Jatuporn Phoemsapthawee

**Affiliations:** 1 Faculty of Allied Health Sciences, Burapha University, Chonburi 20131, Thailand; tadsawiya@go.buu.ac.th (T.P.); orachorn@go.buu.ac.th (O.B.); surachat.bu@go.buu.ac.th (S.B.); nattaphol@go.buu.ac.th (N.P.); 2 Department of Science and Bioinnovation, Faculty of Liberal Arts and Science, Kasetsart University, Nakhon Pathom 73140, Thailand; siriporn.srip@ku.th; 3 Department of Sports Science, Faculty of Sports and Health Science, Kasetsart University, Nakhon Pathom 73140, Thailand; jatuporn.w@ku.th

**Keywords:** high-intensity interval training, asparagus root extract, 20-hydroxyecdysone, oxidative stress, inflammation, metabolic profile, obesity, randomized controlled trial

## Abstract

Excess adiposity is associated with increased oxidative stress and inflammation, which contribute to metabolic dysregulation. Both exercise training and bioactive plant-derived compounds have been explored as therapeutic strategies to mitigate these effects. Asparagus (*Asparagus officinalis* L.) root extract, rich in ecdysteroids such as 20-hydroxyecdysone (20E), exhibits potent antioxidant and anti-inflammatory activities. This randomized controlled trial investigated the combined effects of high-intensity interval training (HIIT) and asparagus root extract (ARE) supplementation on metabolic parameters, oxidative stress, inflammatory biomarkers, and white blood cell counts in overweight and obese adults. Seventy-two participants aged 18–30 years with a body mass index ≥ 23 kg/m^2^ were randomly assigned to one of four groups: control (CON), ARE supplementation only (ARE), HIIT only (HIIT), and combined intervention (COM). The HIIT protocol comprised a modified Tabata regimen of progressive bodyweight intervals at 80–90% and 40–50% of maximal perceived exertion, performed three times per week for 12 weeks. Participants in the ARE and COM groups received a daily oral dose of ARE providing 1.71 ± 0.24 mg/kg/day of 20E. Compared with the CON group, the HIIT group showed significant reductions in total cholesterol (TC), the TC/high-density lipoprotein cholesterol (HDLC) ratio, and blood glucose levels, alongside significant increases in HDLC and superoxide dismutase (SOD) activity (all *p* < 0.05). The COM group demonstrated significant decreases in protein carbonyls and interleukin-6 levels and in the TC/HDLC ratio (all *p* < 0.05) as well as a significant increase in SOD activity (*p* = 0.002). The ARE group, meanwhile, exhibited significant increases in both SOD activity (*p* < 0.001) and malondialdehyde levels (*p* = 0.017). These findings suggest that combining HIIT with ARE supplementation produces synergistic improvements in oxidative and inflammatory status, whereas HIIT alone primarily enhances metabolic regulation in overweight and obese individuals.

## 1. Introduction

Excess adiposity, commonly referred to clinically as overweight or obesity, is a rapidly growing global health problem, with prevalence rates continuing to rise across both developed and developing countries [1]. This condition is widely recognized as a major risk factor for type 2 diabetes mellitus, cardiovascular disease, and nonalcoholic fatty liver disease, among other chronic conditions [2]. Beyond the accumulation of adipose tissue itself, excess adiposity is characterized by systemic low-grade inflammation and oxidative stress, both of which contribute significantly to the onset and progression of metabolic and cardiovascular complications [3]. At the molecular level, caloric excess and adipose tissue expansion promote the overproduction of reactive oxygen species (ROS) through mitochondrial dysfunction and the activation of NADPH oxidases [4]. This redox imbalance is further aggravated by diminished activity of endogenous antioxidant systems, including superoxide dismutase (SOD), catalase (CAT), and glutathione peroxidase (GPx) [5]. Concurrently, adipose tissue-derived cytokines such as tumor necrosis factor-α (TNF-α) and interleukin-6 (IL-6) sustain chronic inflammatory signaling that exacerbates metabolic dysfunction [6].

Physical exercise is one of the most effective non-pharmacological strategies for reducing oxidative stress and inflammation associated with excess adiposity. Among various exercise modalities, high-intensity interval training (HIIT) has emerged as particularly promising because of its time efficiency and robust physiological benefits compared with traditional moderate-intensity continuous training [7]. HIIT involves alternating short bursts of near-maximal effort with periods of active recovery, thereby stimulating mitochondrial biogenesis, improving lipid metabolism, enhancing insulin sensitivity, and upregulating endogenous antioxidant defenses [8]. Accumulating evidence indicates that HIIT can decrease circulating inflammatory cytokines and improve lipid profiles in individuals with excess adiposity [9,10]. However, the extent of its effects on oxidative damage markers and immune cell regulation remains less well understood, suggesting that additional adjunct strategies may be required to optimize outcomes.

In parallel, plant-derived bioactive compounds have attracted increasing interest as dietary supplements for modulating oxidative and inflammatory pathways. Asparagus (*Asparagus officinalis* L.) is a widely consumed vegetable with both nutritional and medicinal value [11]. Its root extract contains high levels of ecdysteroids, particularly 20-hydroxyecdysone (20E), a polyhydroxylated steroid hormone structurally similar to vertebrate steroids but with distinct biological activities [12]. Ecdysteroids have been reported to exert antioxidant effects by scavenging ROS and enhancing enzymatic antioxidant defense systems [13]. They also demonstrate anti-inflammatory activity by suppressing NF-κB activation and reducing pro-inflammatory cytokine release [14]. Furthermore, ecdysteroids may exhibit hypolipidemic effects through lowering plasma cholesterol levels and modulating triglyceride lipase activity [15]. Despite these promising preclinical findings, human studies evaluating asparagus root extract (ARE) supplementation remain limited, and its efficacy in addressing oxidative and inflammatory dysregulation associated with excess adiposity has not been systematically examined.

Given that exercise and plant-derived supplements may act through complementary mechanisms, combining HIIT with ARE supplementation could provide additive or synergistic benefits. Exercise-induced improvements in mitochondrial function and redox signaling may be further reinforced by the antioxidant properties of ecdysteroids, while the anti-inflammatory potential of ARE may complement exercise-mediated reductions in cytokine production. Such interactions could also extend to lipid metabolism and immune cell regulation, thereby addressing multiple excess adiposity-related pathophysiological processes simultaneously.

Therefore, the present study aimed to investigate the combined effects of HIIT and ARE supplementation on key metabolic parameters, including blood glucose, lipid profiles, oxidative stress markers, and inflammatory biomarkers, in overweight and obese adults. In addition, immune cell profiles were assessed through comprehensive white blood cell counts. We hypothesized that the combined intervention would elicit greater improvements than HIIT alone, thereby supporting the potential of integrating structured exercise with targeted nutritional supplementation as an effective lifestyle strategy for managing excess adiposity and its associated metabolic disturbances.

## 2. Results

### 2.1. Baseline Participant Characteristics

**Figure 1:**
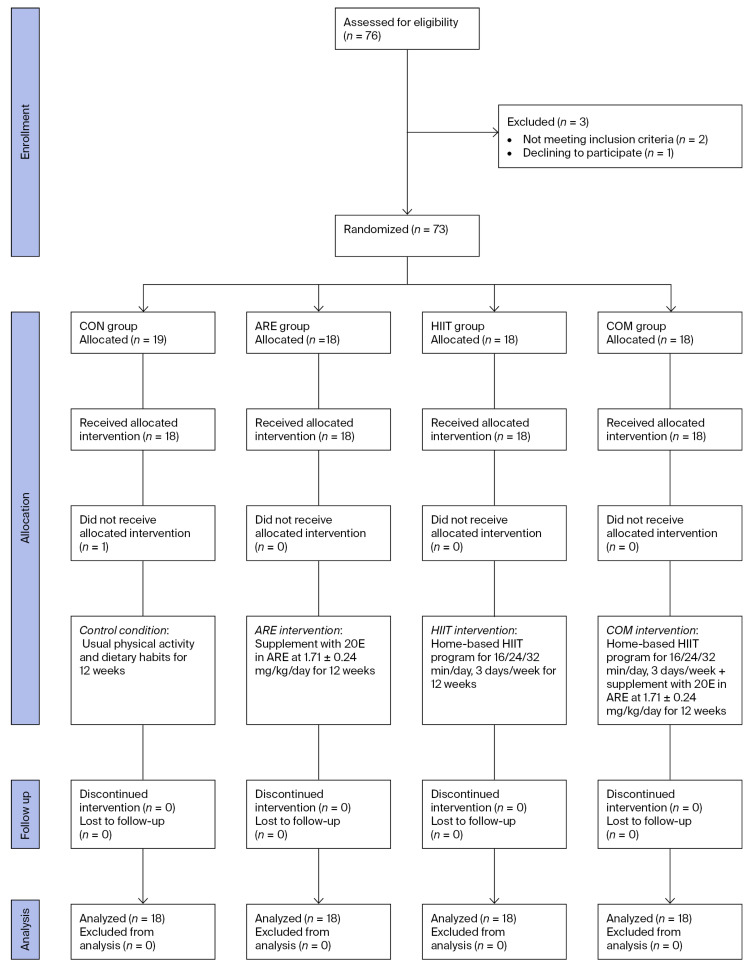
CONSORT flow diagram illustrating participant enrollment, randomization, group allocation, follow-up, and analysis.

A total of seventy-six volunteers were screened for eligibility. Three individuals were excluded for not meeting the inclusion criteria (body mass index [BMI] < 22.9 kg/m^2^; *n* = 2) or declining to participate (*n* = 1). Consequently, seventy-three participants were enrolled, randomized, and allocated into four study groups. Among these, one participant was excluded due to an inability to provide a pretest blood sample. Thus, seventy-two participants (*n* = 18 per group) received the assigned interventions and completed the 12-week protocol. Data from all participants were included in the final analysis (Figure 1).

The study enrolled 72 young adults, comprising 58 females (80.56%) and 14 males (19.44%). A total of 55 participants (76.39%) were classified as obese and 17 (23.61%) as overweight. The mean BMI was 28.19 ± 4.67 kg/m^2^ (range: 23.10–44.90 kg/m^2^), with a mean age of 20.65 ± 1.91 years (range: 18–27 years) and a mean physical activity score of 7.09 ± 1.03 (range: 5.00–9.65), indicating that most participants exhibited an active lifestyle. Baseline characteristics did not differ significantly among the randomized groups (Table 1). Furthermore, no significant changes were observed from baseline to week 12 in BMI, fat-free mass, fat mass, percent body fat, physical activity score, or daily energy intake.

### 2.2. Blood Glucose and Lipid Profile

**Figure 2:**
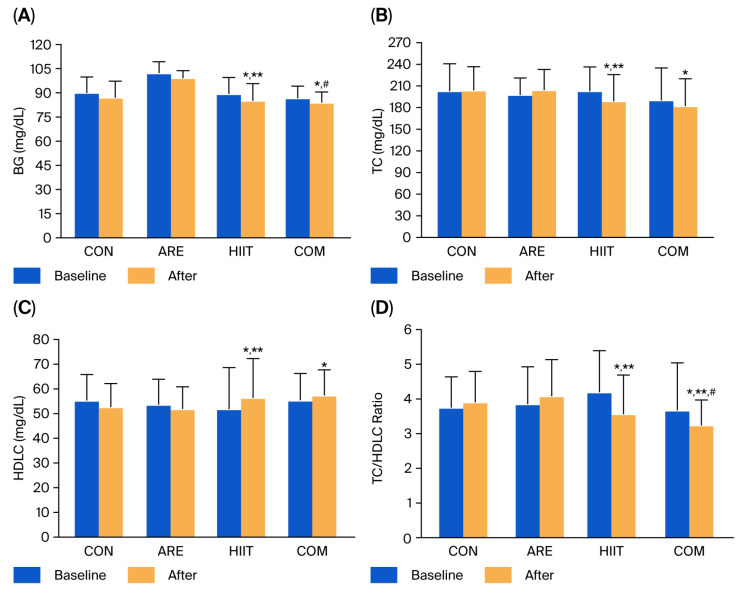
Blood glucose (BG) (**A**), total cholesterol (TC) (**B**), high-density lipoprotein cholesterol (HDLC) (**C**), and TC/HDLC ratio (**D**) in the control (CON), asparagus root extract supplementation (ARE), high-intensity interval training (HIIT), and combined HIIT with ARE supplementation (COM) groups at baseline and after the 12-week intervention. Data are presented as mean ± SD. * *p* < 0.05 vs. before intervention; ** *p* < 0.05 vs. CON group; # *p* < 0.05 vs. ARE group.

At baseline, blood glucose (BG) and lipid profiles—including total cholesterol (TC), triglycerides (TG), high-density lipoprotein cholesterol (HDLC), low-density lipoprotein cholesterol (LDLC), very-low-density lipoprotein cholesterol (VLDLC), TC/HDLC ratio, and LDLC/HDLC ratio—did not differ significantly among the groups. Within-group analyses revealed that BG levels significantly decreased in both the HIIT (−4.39 mg/dL, *p* = 0.016) and COM groups (−2.78 mg/dL, *p* = 0.031) after 12 weeks of intervention. Compared with the CON group, the HIIT group exhibited a significantly greater reduction in BG levels (−2.23 mg/dL, *p* = 0.048). Furthermore, the COM group showed a significantly greater decrease in BG levels (−15.17 mg/dL, *p* = 0.033) compared with the ARE group (Figure 2A).

As shown in Figure 2B–D and Table 2, significant improvements in the lipid profile were observed in both the HIIT and COM groups. Specifically, these groups exhibited marked reductions in TC (−13.89 and −7.83 mg/dL, respectively; *p* = 0.003 and *p* = 0.026, respectively) and the TC/HDLC ratio (−0.63 and −0.43, respectively; *p* < 0.001 and *p* = 0.006, respectively), along with increases in HDLC (+4.56 and +1.95 mg/dL, respectively; *p* = 0.001 and *p* = 0.017, respectively). Conversely, the ARE group showed significant increases in LDLC (+14.06 mg/dL, *p* = 0.006) and the LDLC/HDLC ratio (+0.33, *p* = 0.035). Between-group analyses indicated that the HIIT and COM groups achieved significantly greater reductions in the TC/HDLC ratio (−0.34 and −0.66, respectively; *p* = 0.045 and *p* = 0.049, respectively) compared with the CON group. Moreover, the HIIT group demonstrated significantly greater improvements in TC (−15.34 mg/dL, *p* = 0.029) and HDLC (+3.72 mg/dL, *p* = 0.002) compared with the CON group. Additionally, the COM group exhibited a significantly greater reduction in the TC/HDLC ratio (−0.84, *p* = 0.018) compared with the ARE group.

### 2.3. Oxidative Stress Markers

**Figure 3:**
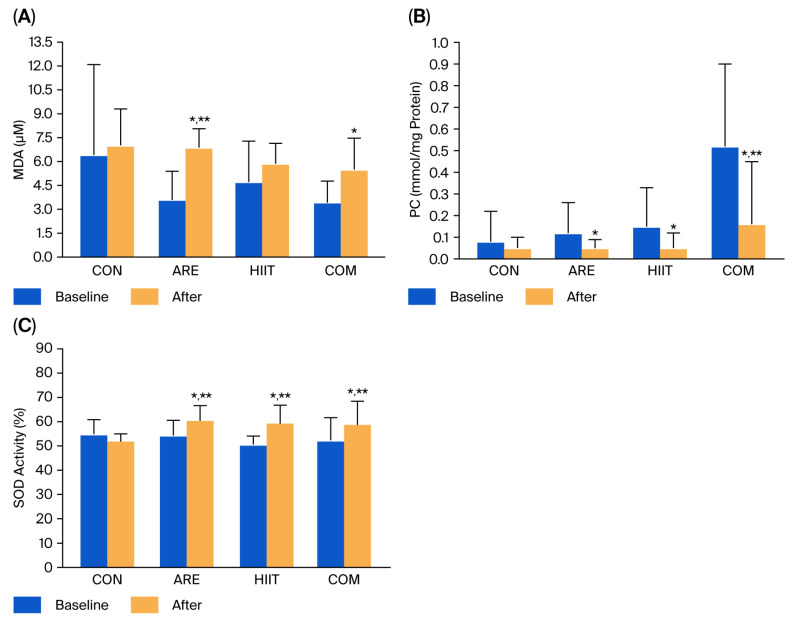
Malondialdehyde (MDA) (**A**), protein carbonyl (PC) (**B**), and superoxide dismutase (SOD) activity (**C**) levels in the control (CON), asparagus root extract supplementation (ARE), high-intensity interval training (HIIT), and combined HIIT with ARE supplementation (COM) groups at baseline and after the 12-week intervention. Data are presented as mean ± SD. * *p* < 0.05 vs. before intervention; ** *p* < 0.05 vs. CON group.

At baseline, significant differences in malondialdehyde (MDA) and protein carbonyl (PC) levels were observed among the CON, ARE, HIIT, and COM groups (*p* < 0.05). Therefore, one-way analysis of covariance with baseline adjustment was applied to assess post-intervention differences among groups. Within-group analysis showed that MDA levels increased significantly after 12 weeks in both the ARE (+3.30 μM, *p* < 0.001) and COM (+2.06 μM, *p* = 0.001) groups. Compared with the CON group, only the ARE group exhibited significantly higher post-intervention MDA concentrations (+2.71 μM, *p* = 0.017), with no significant differences among the intervention groups (Figure 3A).

In contrast, PC levels significantly decreased in all three intervention groups after 12 weeks (ARE: −0.07; HIIT: −0.10; COM: −0.32 nmol/mg protein; all *p* < 0.05). Compared with the CON group, only the COM group demonstrated a significantly greater reduction in PC levels (−0.32 nM/mg protein, *p* = 0.008) (Figure 3B). No significant differences were detected among the intervention groups.

SOD activity significantly increased in all intervention groups following the 12-week period (ARE: +6.10%; HIIT: +8.67%; COM: +6.45%; all *p* < 0.05). Compared with the CON group, participants in the ARE, HIIT, and COM groups exhibited significantly higher post-intervention SOD activity (ARE: +8.49%; HIIT: +7.28%; COM: +6.82%; all *p* < 0.01). No significant differences were observed among the three intervention groups (Figure 3C).

### 2.4. Inflammatory Biomarkers

**Figure 4:**
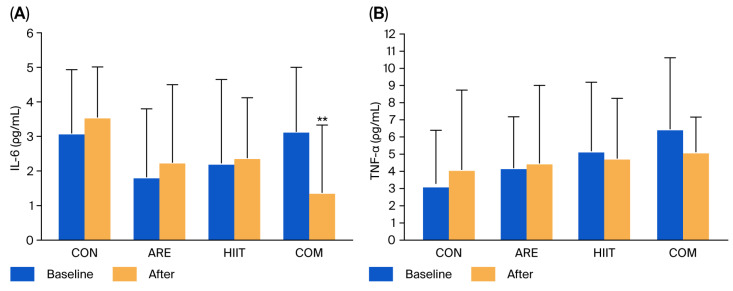
Interleukin-6 (IL-6) (**A**) and tumor necrosis factor-alpha (TNF-α) (**B**) levels in the control (CON), asparagus root extract supplementation (ARE), high-intensity interval training (HIIT), and combined HIIT with ARE supplementation (COM) groups at baseline and after the 12-week intervention. Data are presented as mean ± SD. ** *p* < 0.05 vs. CON group.

At baseline, IL-6 and TNF-α levels did not differ significantly among groups. After the 12-week intervention, no significant changes were observed in either cytokine within any group. Although TNF-α levels remained comparable among groups, IL-6 levels were significantly lower in the COM group compared with the CON group (−1.75 pg/mL, *p* = 0.008) (Figure 4). Similarly to oxidative stress markers, no significant differences in IL-6 or TNF-α levels were found among the intervention groups.

### 2.5. White Blood Cell Counts

**Figure 5:**
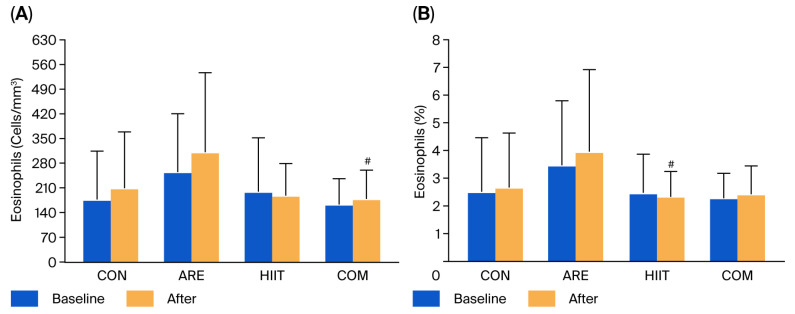
Eosinophils counts (**A**) and percentages (**B**) in the control (CON), asparagus root extract supplementation (ARE), high-intensity interval training (HIIT), and combined HIIT with ARE supplementation (COM) groups at baseline and after the 12-week intervention. Data are presented as mean ± SD. # *p* < 0.05 vs. ARE group.

At baseline, no significant differences were observed in white blood cell (WBC) counts—including total WBCs, neutrophils, eosinophils, basophils, lymphocytes, and monocytes—among the four groups, either in absolute numbers or percentages, except for basophil counts, which were significantly lower in the COM group compared with the ARE group. After the 12-week intervention, eosinophil counts were significantly lower in the COM group than in the ARE group (−132.11 cells/mm^3^; *p* = 0.033). Similarly, the eosinophil percentage was significantly lower in the HIIT group compared with the ARE group (−1.61%; *p* = 0.041) (Figure 5). No significant differences were observed in other WBC subtypes within or among the four groups (Table 3).

### 2.6. Liver Function Parameters

To assess the potential adverse effects of the 12-week ARE supplementation in the ARE and COM groups, liver function parameters, including alanine aminotransferase (ALT) and aspartate aminotransferase (AST), were evaluated. Following the 12-week interventions, AST and ALT levels showed no significant changes from baseline in either group. These findings indicate that 12-week ARE supplementation was well tolerated and did not adversely affect liver function in overweight and obese participants (Table 2).

## 3. Discussion

After 12 weeks, both the HIIT and COM interventions produced significant reductions in BG, whereas no changes were observed in the ARE and CON groups. The BG reduction observed in the HIIT group aligns with previous evidence indicating that high-intensity exercise enhances glucose homeostasis through multiple mechanisms, including increased GLUT4 translocation, activation of AMPK, and improved mitochondrial biogenesis and oxidative capacity, thereby enhancing insulin sensitivity in overweight individuals [16,17,18]. Notably, the COM group exhibited the greatest decrease in BG, suggesting a synergistic interaction between HIIT and ARE supplementation. Asparagus phytochemicals such as quercetin, rutin, and saponins possess antioxidant, anti-inflammatory, and insulin-sensitizing properties that may potentiate HIIT-induced AMPK activation and GLUT4 upregulation, resulting in additive improvements in glucose regulation [19,20,21].

HIIT also elicited favorable alterations in lipid metabolism, characterized by reductions in TC and the TC/HDLC ratio, accompanied by an elevation in HDLC. These outcomes are consistent with previous findings that HIIT enhances lipid turnover by stimulating lipoprotein lipase activity, activating PPAR-α/AMPK signaling pathways, increasing fatty acid oxidation, and promoting reverse cholesterol transport [22,23,24]. In contrast, ARE supplementation alone unexpectedly increased LDLC and the LDLC/HDLC ratio. Although asparagus-derived bioactives have been reported to inhibit HMG-CoA reductase and facilitate bile acid excretion, such effects may depend on longer intervention periods, higher compound bioavailability, or the presence of concurrent metabolic stress.

In interpreting the present findings, it is also important to consider the potential influence of both supplementation dosage and exercise load. The ARE dosage used in this study (providing 1.71 ± 0.24 mg/kg/day of 20E) was selected based on prior experimental work; however, it is possible that this level was insufficient to produce measurable metabolic benefits in the absence of concurrent metabolic activation from exercise. Dose–response relationships for ecdysteroid-rich extracts in humans remain poorly characterized, and higher or periodized dosing strategies may be required to elicit more robust lipid-modulating or antioxidant effects. Similarly, although the HIIT protocol employed a progressive increase in training load across the 12-week period, variations in session duration, interval structure, or overall weekly training volume may lead to different oxidative and inflammatory adaptations. Future studies should therefore systematically evaluate both supplement dose and training load—independently and in combination—to determine the optimal parameters for maximizing metabolic, redox, and inflammatory outcomes.

Notably, although a previous review demonstrated the safety of *Asparagus officinalis* L. extracts at doses up to 500 mg/kg [25], it did not quantify 20E or other ecdysteroid concentrations, limiting direct comparison with our dosage approach based on standardized 20E content. Given the considerable variability in phytochemical profiles across asparagus preparations, future studies should report both extract mass and quantified bioactive constituents to facilitate more accurate dose–response evaluation.

Importantly, the COM group demonstrated lipid improvements comparable to those induced by HIIT alone, with a significantly greater reduction in the TC/HDLC ratio relative to the ARE group. This observation aligns with previous trials showing enhanced cardiometabolic benefits when aerobic exercise is paired with polyphenol-rich supplementation, such as pomegranate, Spirulina, or curcumin [19,26,27]. Notably, baseline BG and lipid parameters in our participants were within normal reference ranges; therefore, the observed changes should be interpreted as favorable physiological shifts rather than clinically meaningful corrections. These adaptations likely represent early metabolic responses to the interventions rather than treatment of pathological abnormalities.

The unexpected increases in LDLC and MDA observed in the ARE-only group suggest that ARE supplementation may not exert favorable lipid or oxidative effects when administered without concurrent metabolic activation. These findings raise the possibility that certain constituents within the extract may behave differently under low metabolic demand, or that the administered dose may have induced an adaptive oxidative response that did not resolve within the 12-week period. Therefore, the effects of ARE alone should be interpreted with caution. Future studies should examine dose–response relationships and determine whether exercise-induced metabolic stress is required to unlock the beneficial actions of ARE.

In contrast, the rise in MDA observed in the COM group may reflect an adaptive oxidative adaptation rather than oxidative injury, as mild increases in lipid peroxidation are known to trigger upregulation of endogenous antioxidant defenses during the early phase of metabolic adaptation [28,29,30]. Consistent with this interpretation, SOD activity increased across all intervention groups, indicating enhanced enzymatic antioxidant capacity. This adaptation parallels the changes seen in PC, which decreased significantly in all intervention groups—most prominently in the COM group—suggesting improved protein redox balance. These findings likely reflect synergistic actions of HIIT and ARE, in which HIIT-driven AMPK–PGC-1α–Nrf2 signaling promotes endogenous antioxidant defenses, while asparagus-derived polyphenols modulate NF-κB/MAPK pathways and directly scavenge ROS. Together, these complementary mechanisms appear to maximize protection against protein oxidation, aligning with the established pattern in which early oxidative stress during training normalizes as antioxidant capacity strengthens [28,31].

Neither HIIT nor ARE alone significantly altered IL-6 or TNF-α levels, likely due to the participants’ mild baseline inflammation and the relatively short intervention duration. Although HIIT can acutely elevate IL-6 during exercise bouts, chronic training generally exerts anti-inflammatory effects by reducing adiposity and enhancing antioxidant enzyme activity [32,33]. Polyphenol supplementation alone typically produces modest cytokine changes unless combined with metabolic activation or administered at higher doses (e.g., resveratrol ≥ 500 mg/day), as demonstrated in randomized controlled trials and meta-analyses of quercetin, green tea catechins, and other phytochemicals [34,35,36]. As summarized in previous systematic reviews [37,38,39], the impact of polyphenols on IL-6 modulation remains inconsistent in the absence of exercise. In agreement with these findings, only the COM group exhibited a significant reduction in IL-6 compared with the CON group, suggesting that the combined redox and anti-inflammatory stimuli more effectively attenuated low-grade inflammation [40]. Regular exercise promotes the release of myokines such as IL-10 and IL-1Ra, contributing to an anti-inflammatory milieu through suppression of NF-κB signaling [33,41]. Likewise, plant extracts from the genus *Asparagus* have been shown in vitro to inhibit NF-κB activation and nitric oxide release, supporting a mechanistic basis for their anti-inflammatory activity [42,43].

The absence of significant TNF-α changes in our study likely reflects differences in baseline inflammation and exercise workload compared with the previous study [44]. Given that TNF-α typically responds more robustly to higher training volumes and exhibits substantial inter-individual variability in metabolically healthy populations, the large effect reported previously may be study-specific. Our findings align with prior evidence indicating that modest, low-volume, or short-duration exercise often produces small or inconsistent changes in TNF-α, particularly among healthy or moderately active individuals [45,46].

Total WBC counts remained stable across all groups, indicating that neither the HIIT protocol nor ARE supplementation induced immune suppression or leukocytosis and confirming that the training stimulus was well tolerated. Notably, eosinophil counts significantly decreased in the HIIT and COM groups compared with the ARE group. Although eosinophils are not traditionally considered primary mediators of excess adiposity-related inflammation, emerging evidence indicates that they play a key role in immune-metabolic regulation by sustaining alternatively activated macrophages (M2 macrophages) within adipose tissue and promoting anti-inflammatory signaling and glucose homeostasis [47,48]. Therefore, the observed reduction in eosinophils may reflect a shift toward improved systemic immune balance and attenuation of low-grade inflammation in response to exercise-based interventions. This interpretation aligns with previous findings that moderate-to-high intensity interval exercise can reduce eosinophil-associated inflammatory activity in both airway disease and systemic inflammatory contexts, likely via down-regulation of Th2-IL-5 signaling pathways [32,49,50,51]. Reporting both absolute eosinophil counts and percentages allowed for a more comprehensive assessment of leukocyte distribution, as exercise can transiently alter total WBC counts and thereby influence relative proportions. The ARE group did not demonstrate a similar reduction in eosinophils, which may reflect the mild immunomodulatory properties of phytochemicals, although this effect was not clinically significant.

Collectively, our findings indicate that HIIT served as the primary driver of improvements in glucose regulation and lipid metabolism. Although the combined HIIT + ARE intervention produced favorable metabolic, oxidative, and inflammatory outcomes, these effects were not substantially greater than those achieved through HIIT alone. In contrast, ARE supplementation in isolation did not yield metabolic or inflammatory benefits and was associated with increases in LDLC and MDA, despite elevating SOD activity. This pattern suggests that ARE may not exert favorable physiological effects in the absence of concurrent metabolic activation induced by exercise. These observations underscore the need for caution when considering ARE as a standalone supplement and highlight the importance of evaluating factors such as dose, bioactive composition, and the potential synergistic interaction between supplementation and exercise.

Finally, ALT and AST remained stable in the ARE and COM groups, indicating that ARE at this dose was safe and did not impose hepatic burden. Although post-intervention ALT values exhibited a wider SD, this variability was attributable to four participants (ARE: *n* = 2; COM: *n* = 2) whose ALT levels were already slightly above the clinical reference range at baseline (ALT < 40 U/L) [52] and who all had BMI values > 24.9 kg/m^2^—an adiposity profile commonly associated with mild ALT elevation [53]. Importantly, none of these individuals demonstrated a worsening of ALT; rather, values were either stable or improved compared with baseline (ARE: from 74 to 39 U/L and from 54 to 54 U/L; COM: from 68 to 43 U/L and from 52 to 45 U/L). No new ALT elevations were observed, confirming that the increased post-intervention variability reflects pre-existing hepatic heterogeneity rather than any treatment-related effect. Collectively, the findings support HIIT as an effective strategy for improving glycemic and lipid profiles and demonstrate that combining HIIT with ARE amplifies metabolic, redox, and inflammatory benefits without adverse hepatic effects.

This study has several limitations that should be acknowledged. First, although the sample size was adequate to detect moderate effects, it may have been underpowered to identify small but biologically meaningful changes in inflammatory or immunological parameters. Second, overweight and obese participants were analyzed as a single elevated-BMI cohort. Although baseline BMI and the distribution of overweight versus obese individuals did not differ significantly across groups, this approach may mask potential differences in metabolic, inflammatory, and exercise-related responses that are known to vary across BMI categories. The study was underpowered to conduct meaningful stratified analyses by BMI class; therefore, future research with larger sample sizes should incorporate BMI-stratified or obesity-class analyses to better delineate differential physiological responses to HIIT and ARE supplementation. Third, the study population consisted primarily of young, active women, which limits the generalizability of the findings to older adults, sedentary individuals, men, or individuals with more severe metabolic impairments. Because physiological responses to high-intensity exercise and phytochemical supplementation may vary across demographic and clinical subgroups, the uneven distribution of female participants across groups may also influence metabolic, inflammatory, and exercise-related responses. Although statistical testing indicated no significant differences in sex distribution between groups, this imbalance may still restrict external validity. Future studies should aim to recruit more balanced or sex-stratified samples to strengthen generalizability. Fourth, many participants were classified as ‘active’ or ‘athletic’ at baseline despite being overweight/obese, reflecting the lifestyle characteristics of the university student population. This relatively high baseline activity level may have blunted the magnitude of training or supplementation effects and limits the generalizability of the findings to sedentary or clinically at-risk populations. Fifth, although the discussion references potential molecular mechanisms (e.g., AMPK, Nrf2, NF-κB) to contextualize the findings, these pathways were not directly measured in this study. Therefore, these explanations remain speculative, and future studies employing molecular analyses such as muscle biopsies or protein expression assays are required to confirm the proposed mechanisms. Sixth, although ecologically valid, dietary intake and habitual physical activity outside the intervention were self-reported, making them susceptible to recall bias and day-to-day variability. Despite the use of standardized questionnaires, these factors may have introduced residual confounding. Future studies should incorporate objective monitoring tools—such as weighed food records, photographic food logs, digital dietary-tracking applications, accelerometers, or wearable activity trackers—to improve measurement accuracy and minimize reporting bias. Seventh, variations in adiposity levels and fat distribution may act as confounding factors, as adipose tissue quantity and regional deposition can influence inflammatory and redox responses. In this study, adiposity was assessed using a bioelectrical impedance analysis device, which does not differentiate total adipose tissue or regional fat distribution with the same precision as dual-energy X-ray absorptiometry (DEXA) or BodPod analysis. This limitation may affect the interpretation of adiposity-related outcomes. Future studies should incorporate more accurate body composition methods—such as DEXA, BodPod, or other validated modalities—to better characterize adiposity and its potential moderating effects. Eighth, although fasting BG was assessed using a standardized hexokinase assay, HbA1c was not measured. HbA1c provides an integrated indicator of average glycemic exposure over the previous 2–3 months and is clinically important in evaluating chronic glucose regulation. Its absence limits the ability to determine whether the interventions produced longer-term glycemic changes beyond the fasting state. Future studies should incorporate HbA1c to complement BG measurements and strengthen metabolic interpretations. Ninth, plasma insulin was not assessed, which precluded the calculation of insulin resistance indices such as HOMA-IR. Although fasting BG provides useful information on short-term glycemic status, the absence of insulin measurements limits the ability to comprehensively evaluate metabolic regulation. Future studies should incorporate fasting insulin or dynamic measures of insulin sensitivity to strengthen mechanistic interpretations. Tenth, eosinophils are not the most specific biomarker for excess adiposity-related inflammation, which is more accurately characterized by macrophage infiltration, M1/M2 macrophage polarization, neutrophil activation, and cytokine dynamics. Although eosinophil changes may reflect shifts in systemic immune tone, they provide only a partial representation of the inflammatory processes associated with metabolic dysfunction. Thus, future studies should incorporate a broader panel of mechanistic immunological markers, such as macrophage phenotypes, neutrophil subsets, and pro- and anti-inflammatory cytokines, to more precisely elucidate the immunometabolic pathways influenced by HIIT and ARE supplementation. Finally, this study was conducted as a non-blinded randomized controlled trial. Because participants were aware of their group assignments, especially for the HIIT intervention, performance bias related to differential motivation cannot be fully excluded. In addition, the absence of a placebo supplement for the ARE group may have introduced detection bias, as outcome assessors were not fully blinded to group allocation. Although standardized laboratory procedures and independent monitoring were implemented to minimize these risks, a double-blind, placebo-controlled design would more rigorously isolate the effects of ARE supplementation and should be employed in future investigations.

Additionally, the elevations in LDLC and MDA observed in the ARE-only group underscore the need to more clearly delineate the isolated physiological effects of ARE supplementation. In the absence of molecular or mechanistic data, it remains unclear whether these changes reflect dose-dependent responses, interactions among extract constituents, or the lack of exercise-induced metabolic stimulation that may be required to activate the putative beneficial pathways of ARE. To address these uncertainties, future studies should incorporate multi-omics platforms—such as transcriptomics, proteomics, and metabolomics—to elucidate the mechanistic signatures of ARE supplementation and its interactions with exercise stimuli.

Moreover, investigating sex- and age-specific responses may help identify biological moderators of efficacy. Comparative trials examining distinct exercise modalities (e.g., HIIT versus moderate continuous training) in combination with plant-derived antioxidants would further clarify synergistic or divergent physiological effects. Evaluating clinically relevant endpoints, including endothelial function, vascular stiffness, and comprehensive cardiometabolic risk markers, is also warranted. Lastly, longer-term follow-up studies are necessary to determine whether the short-term oxidative adaptations observed here translate into sustained metabolic resilience and durable cardiometabolic health benefits.

## 4. Materials and Methods

### 4.1. Study Design and Sample Size

This randomized controlled trial was conducted in Mueang District, Chonburi Province, Thailand. A total of 76 overweight (BMI 23.0–24.9 kg/m^2^) and obese (BMI > 24.9 kg/m^2^) volunteers were recruited in September 2022. The sample size was estimated based on data from a previous study by Soltani et al. [44], which examined the effects of a 10-week combined all-extremity HIIT program on inflammatory cytokines in obese young females. In that study, the mean difference in TNF-α levels between the experimental and control groups was 16.87, with a standard deviation of 13.57. Using G*Power software version 3.1.9.4, with a significance level of 0.05 and statistical power of 0.80, the required sample size was calculated to be 18 participants per group. Allowing for an anticipated 10% dropout rate, the total required sample size was set at a minimum of 72 participants.

### 4.2. Ethical Considerations and Informed Consent

This study was conducted in accordance with the principles of the Declaration of Helsinki and approved by the Burapha University Institutional Review Board (approval no. G-HS018/2565; approval date: 24 May 2022). Participants were provided with and signed an informed consent form before screening. The trial was retrospectively registered at the Thai Clinical Trials Registry (Identifier: TCTR20230608004; registration date: 8 June 2023).

### 4.3. Screening of Participants

Participant screening was conducted using both physical and psychological health questionnaires, accompanied by a comprehensive physical examination that included anthropometric measurements and vital sign assessments. Participants completed a detailed interview regarding current and past use of medications or dietary supplements that could affect metabolic, inflammatory, or oxidative stress markers to ensure adherence to the exclusion criteria. Eligible participants were males or females aged 18–30 years with a BMI greater than 22.9 kg/m^2^. According to the Asia–Pacific classification, individuals with a BMI of 23.0–24.9 kg/m^2^ were categorized as overweight, while those with a BMI exceeding 24.9 kg/m^2^ were classified as obese. Exclusion criteria included the regular use of medications or dietary supplements known to influence body weight, glucose metabolism, lipid profiles, liver enzymes, inflammatory markers, or oxidative stress. These included, but were not limited to, antidiabetic agents (e.g., metformin, insulin), lipid-lowering drugs (e.g., statins, fibrates), anti-inflammatory agents, antioxidant supplements, probiotics, herbal supplements with metabolic effects, or any over-the-counter formulations targeting weight loss or metabolic enhancement; known allergies to foods—particularly shoots or bulbous plants such as asparagus, bamboo shoots, green onions, onions, leeks, garlic, or chives—and hypersensitivity to drugs, including lithium-based compounds (e.g., lithium carbonate). Participants were also excluded if they were pregnant or breastfeeding, smoked habitually (>30 packs per year), consumed alcohol regularly (>1 cup per day), or had a history of substance use or any cardiovascular, hepatic, renal, musculoskeletal, infectious, oncologic, neurological, or psychiatric disorder.

To ensure that the results were minimally influenced by other confounding factors, including potential changes in daily physical activity and dietary intake, participants were instructed not to modify their routine physical activities or dietary habits throughout the study period. The 24 h dietary recall (24HR) and Food Frequency Questionnaire (FFQ) were used to assess participants’ dietary intake behaviors [54]. Participants were asked to record their dietary intake for 3 days per week, including 2 weekdays and 1 weekend day. These assessments were conducted one week before the pretest to establish baseline dietary intake and again at the 12th week of the study period. Daily energy intake was calculated in kilocalories per day and normalized to kilocalories per day per kilogram of body weight. Habitual physical activity was assessed using the Baecke Habitual Physical Activity Questionnaire (Thai version), which consists of three domains (work, sport, and leisure activities), each scored on a 5-point Likert scale. The total physical activity score was calculated as the sum of the three indices (range: 3–15), with higher scores indicating greater habitual activity. Based on validated Thai cut-points, physical activity levels were classified as sedentary (<6.0), active (6.0–9.0), or athletic (>9.0). The Thai version demonstrates good reliability (*r* = 0.76–0.84) and strong construct validity. This scoring system was used to compare baseline activity levels across groups [55,56]. BMI and body composition (fat-free mass, fat mass, percent body fat) were measured by a bioelectrical impedance analyzer (InBody270, InBody Co., Ltd., Daejeon, Republic of Korea).

### 4.4. Experimental Procedures

#### 4.4.1. Randomization and Blinding

A stratified block randomization method was employed to allocate 73 participants into one of four groups: CON, ARE, HIIT, or the COM. Random sequences were generated using the RAND function in Microsoft Excel. This study was conducted as a non-blinded trial. The same researcher (T.P.) performed participant screening, randomization, group allocation, data collection, and data analysis. To minimize potential measurement and procedural bias, another researcher (P.P.) independently monitored and verified all procedures to ensure methodological rigor.

#### 4.4.2. Experimental Protocols

Participants were instructed to fast for at least 8 h and to abstain from alcohol consumption and smoking for 24 h prior to each testing session. They were also required to obtain a minimum of 6 h of sleep and to refrain from performing strenuous exercise for 72 h before both the pretest and posttest assessments.

**Figure 6:**
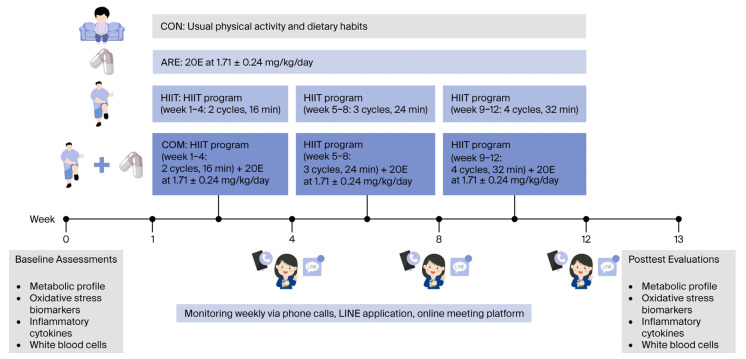
Schematic representation of the study procedures, outlining the sequence of baseline assessments, intervention phases, and posttest evaluations.

Of the 73 allocated participants, 72 received the assigned interventions; one was excluded due to inability to provide a pretest blood sample. Each group (*n* = 18) completed a 12-week intervention according to the assigned condition (Figure 6). The CON maintained their usual physical activity and dietary habits. The HIIT group performed a supervised, home-based HIIT program three times weekly. The COM followed the same HIIT protocol and concurrently received ARE supplementation in capsule form, providing 20E at a dose of 1.71 ± 0.24 mg/kg/day, taken daily after meals (one or two capsules with breakfast and two with dinner). To examine the independent effect of ARE, a separate group (ARE) received the same supplementation regimen without participating in HIIT. Participants consumed the ARE supplement as one or two capsules with breakfast and two capsules with dinner, ensuring a consistent total daily dose across individuals. This structured flexibility was permitted because the primary bioactive compound (20E) demonstrates rapid absorption and comparable systemic exposure whether taken as a single or divided dose. Allowing this dosing variation supported participant adherence without affecting total daily intake [57].

Compliance and adherence to both the HIIT program and ARE supplementation were monitored weekly by the researchers via phone calls, the Line application, and an online meeting platform. Participants who experienced adverse symptoms or serious adverse events potentially related to the interventions (e.g., hospitalization due to exercise or supplementation) or who voluntarily withdrew were immediately excluded from the study and referred for appropriate medical follow-up.

### 4.5. HIIT Program

Tabata-style functional HIIT was implemented in this study, with minor modifications aimed at minimizing muscle fatigue and injury while promoting participant adherence. The training protocol consisted of four functional movements—squat jumps with toe touches, alternating reverse lunges, mountain climbers, and burpees with toe touches—targeting the primary lower limb muscles (quadriceps, gastrocnemius), core stabilizers (transversus abdominis, multifidus), and upper limb muscles (biceps, triceps, pectoralis), as previously described by our group [58]. Each exercise session comprised 4 min Tabata cycles alternating between 20 s of maximal effort and 10 s of rest, followed by an additional 4 min of active recovery, resulting in a total duration of 8 min per cycle. The program was progressively intensified throughout the 12-week intervention, beginning with two cycles per session during weeks 1–4, three cycles during weeks 5–8, and four cycles during weeks 9–12. Exercise intensity was maintained at 80–90% of maximal perceived exertion, while active recovery periods involved light arm-swing movements performed at 40–50% intensity.

### 4.6. ARE Supplement

ARE was prepared by the Faculty of Liberal Arts and Science, Kasetsart University, Nakhon Pathom, Thailand. Following our previously described protocol [59], hard stems of *Asparagus officinalis* L. roots were thoroughly washed, subjected to ultrasonic cleaning for 10 min, cut into 5 mm pieces, and oven-dried at 60 °C for 30 h until a constant weight with approximately 5% moisture content was achieved. The dried material was ground into powder and extracted with 95% ethanol for 3 days. The resulting extract was filtered and evaporated to dryness, and the residue was reconstituted in distilled water for analysis. High-performance liquid chromatography (HPLC) was conducted using a C18 Sep-Pak cartridge (Agilent Technologies, Waldbronn, Germany) at 40 °C with a flow rate of 1 mL/min and a mobile phase consisting of acetonitrile/water (20:80, *v*/*v*). The major ecdysteroid, 20E, was identified based on its UV absorbance at 245 nm, retention time, and spectral matching with authentic standards [12]. The dried extract was encapsulated under aseptic conditions, with each capsule weighing 500 mg and containing 32.2 mg of 20E per g of dry weight. All capsules were stored at −20 °C until use in subsequent experiments.

Moreover, gas chromatography–mass spectrometry (GC–MS) analysis was performed using an Agilent system (Agilent Technologies, Santa Clara, CA, USA) at Central Laboratory (Thailand) Co., Ltd., to identify the chemical constituents of the ARE. The results revealed that 2,3-dihydro-3,5-dihydroxy-6-methyl-4H-pyran-4-one was the predominant compound, accounting for 53.47% of the total peak area. Minor components included benzene, 1-chloro-2-methyl (0.92%), lignocaine (local anesthetic; 6.67%), pentadecanoic acid, 14-methyl-, methyl ester (2.79%), hexadecenoic acid methyl ester (4.39%), ethyl tridecanoate (1.03%), 9,12-octadecadienoic acid (Z,Z)-, methyl ester (3.77%), 10-octadecenoic acid, methyl ester (0.57%), 16-methyl-heptadecanoate (0.87%), and 9,12-octadecadienoic acid, ethyl ester (2.32%).

### 4.7. Study Outcomes and Measurements

#### 4.7.1. Oxidative Stress Biomarker Analysis

Blood samples were collected in the morning after an 8 h overnight fast using standard venepuncture techniques. Lipid peroxidation was determined by measuring MDA concentration in plasma using the thiobarbituric acid reactive substances (TBARS) assay, as described previously [60]. Briefly, 150 μL of plasma was mixed with trichloroacetic acid (TCA, 10%), ethylenediaminetetraacetic acid (5 mM), sodium dodecyl sulfate (8%), and butylated hydroxytoluene (0.5 μg/mL), followed by TBA (0.6%) and incubation in boiling water for 30 min. The samples were then centrifuged at 3500 rpm for 5 min at 4 °C (Eppendorf Centrifuge 5810R, Hamburg, Germany), and the absorbance of the supernatant was measured at 532 nm using a UV/Vis Basic BioSpectrometer (Eppendorf, Hamburg, Germany). A standard curve was constructed using 1,1,3,3-tetraethoxypropane (0.1–10 μmol/L).

Protein oxidation was assessed by determining PC content using the dinitrophenylhydrazine (DNPH) assay, as previously described [61]. In brief, diluted plasma samples were incubated with DNPH in 3.6 M hydrochloric acid (HCl) for 1 h. Proteins were then precipitated with TCA, collected, and repeatedly washed with TCA to remove unreacted DNPH. The resulting protein pellets were dissolved in 6 M guanidine HCl, and absorbance was measured at 360 nm using a UV/Vis Basic BioSpectrometer (Eppendorf, Hamburg, Germany). PC concentration was calculated after subtracting HCl-treated blanks, using a molar extinction coefficient of 22,000 M^−1^·cm^−1^.

Serum SOD activity was quantified using a SOD Assay Kit-WST (Dojindo Laboratories, Kumamoto, Japan) following the manufacturer’s instructions. In brief, 200 μL of WST working solution and 20 μL of enzyme solution were added to each well of a 96-well microplate. The plate was then incubated at 37 °C for 20 min, after which the absorbance was measured at 450 nm using a microplate reader (SpectraMax^®^ ABS, Molecular Devices, San Jose, CA, USA). SOD activity, expressed as an inhibition rate (%), was calculated using the following formula: SOD activity (%) = ((A_blank 1_ − A_blank 3_) − (A_sample_ − A_blank 2_))/(A_blank 1_ − A_blank 3_) × 100.

#### 4.7.2. Inflammatory Cytokine Analysis

Serum concentrations of IL-6 and TNF-α were quantified using commercially available enzyme-linked immunosorbent assay (ELISA) kits (Cat. No. 3460-1HP-2, ELISA Pro: Human IL-6; and Cat. No. 3512-1HP-2, ELISA Pro: Human TNF-α; Mabtech, Stockholm, Sweden), following the manufacturers’ instructions. Optical density was measured at 450 nm using a microplate spectrophotometer (SpectraMax ABS, Molecular Devices LLC, San Jose, CA, USA).

#### 4.7.3. WBC and Metabolic Profile Analysis

WBC counts, including total WBCs, neutrophils, lymphocytes, monocytes, eosinophils, and basophils, as well as BG and lipid profile parameters—TC, TG, LDLC, VLDLC, and HDLC—were determined according to standard clinical laboratory protocols (National Healthcare Systems Co., Ltd., Chonburi, Thailand). Fasting venous blood samples were collected into sodium fluoride tubes to obtain plasma for glucose analysis. Plasma glucose concentrations were determined using an enzymatic hexokinase-based assay following standard clinical laboratory protocols [62]. The TC/HDLC and LDLC/HDLC ratios were subsequently calculated. In addition, to evaluate the potential adverse effects of the 12-week ARE supplementation in the ARE and COM groups, liver function parameters, including ALT and AST, were analyzed.

### 4.8. Statistical Analysis

Data were analyzed using SPSS software (version 26.0; IBM Corp., Armonk, NY, USA) and are presented as mean ± SD. The Kolmogorov–Smirnov test was applied to evaluate the normality of data distribution. Between-group differences among the four groups (CON, ARE, HIIT, and COM) were analyzed using one-way analysis of variance (ANOVA) or analysis of covariance (ANCOVA) when baseline differences were detected, with Bonferroni post hoc correction for multiple comparisons. Within-group comparisons (pre- vs. post-intervention) were conducted using paired *t*-tests. Statistical significance was set at *p* < 0.05.

## 5. Conclusions

This randomized controlled trial demonstrated that the combination of a 12-week HIIT program and *Asparagus officinalis* L. root extract supplementation elicited favorable metabolic, oxidative, and immunological adaptations in overweight and obese adults. Specifically, both the HIIT and COM interventions significantly reduced fasting BG levels, while improvements in TC, HDLC, and the TC/HDLC ratio were observed primarily in the HIIT and COM groups. These metabolic benefits were accompanied by enhanced redox homeostasis, as reflected by increased SOD activity and decreased PC levels. Furthermore, the combined intervention induced modest reductions in IL-6 concentrations and eosinophil counts, suggesting the synergistic attenuation of oxidative and inflammatory stress.

Importantly, the present findings indicate that *A. officinalis* root extract should not be recommended as a standalone strategy for metabolic improvement. Supplementation alone produced neutral effects on glucose regulation and inflammatory markers and was associated with increases in LDLC and MDA, suggesting that its potential physiological benefits may depend on concurrent exercise-induced metabolic activation. Accordingly, *A. officinalis* root extract may be more appropriately considered a complementary nutraceutical rather than an independent intervention. When integrated with structured exercise programs, it may contribute to a safe, non-pharmacological approach for improving glycemic control, lipid regulation, and systemic redox balance, thereby supporting the prevention of metabolic syndrome and related cardiometabolic disorders.

## Figures and Tables

**Table 1:** Baseline and post-intervention characteristics of participants in the control (CON), asparagus root extract supplementation (ARE), high-intensity interval training (HIIT), and combined HIIT with ARE supplementation (COM) groups.

Characteristics	CON (*n* = 18)	ARE (*n* = 18)	HIIT (*n* = 18)	COM (*n* = 18)
Male/female, *n* (%)	5 (28)/13 (72)	2 (11)/16 (89)	5 (28)/13 (72)	2 (11)/16 (89)
Age (years)	21.61 ± 2.06	20.22 ± 1.93	20.72 ± 1.32	20.06 ± 2.01
Overweight/obese, *n* (%)	2 (11)/16 (89)	5 (28)/13 (72)	5 (28)/13 (72)	5 (28)/13 (72)
Physical activity level				
- Sedentary, *n* (%)	3 (17)	3 (17)	3 (17)	1 (6)
- Active, *n* (%)	11 (61)	9 (50)	12 (66)	14 (78)
- Athletic, *n* (%)	4 (22)	6 (33)	3 (17)	3 (17)
BMI (kg/m^2^)				
- Baseline	29.10 ± 5.11	26.98 ± 2.97	29.13 ± 5.48	26.92 ± 2.50
- After	29.10 ± 4.87	27.44 ± 3.04	29.59 ± 5.65	27.19 ± 2.32
Fat-free mass (kg)				
- Baseline	46.04 ± 9.06	44.73 ± 9.60	46.85 ± 9.84	44.12 ±6.50
- After	45.82 ± 9.06	45.11 ± 10.12	47.24 ± 10.20	43.77 ± 6.28
Fat mass (kg)				
- Baseline	31.36 ± 10.23	26.70 ± 6.21	30.79 ±11.64	26.34 ± 5.26
- After	31.53 ± 9.62	27.60 ± 6.72	31.65 ± 11.85	27.42 ± 4.84
Percent body fat				
- Baseline	40.07 ± 7.38	37.44 ± 5.53	39.02 ± 8.98	37.37 ± 5.79
- After	40.44 ± 7.14	38.04 ± 5.64	39.54 ± 9.21	38.52 ± 5.10
Daily energy intake (kcal/day)				
- Baseline	2156 ± 218	2385 ± 215	2439 ± 218	2100 ± 150
- After	2326 ± 204	2043 ± 200	2334 ± 200	2274 ± 119
Daily energy intake (kcal/day/kg)				
- Baseline	28.86 ± 3.63	33.26 ± 2.89	36.13 ± 3.65	30.48 ± 2.14
- After	31.30 ± 3.57	28.75 ± 2.87	34.26 ± 2.54	33.36 ± 2.22
Physical activity score				
- Baseline	7.01 ± 1.07	7.46 ± 1.19	7.04 ± 0.99	6.86 ± 0.85
- After	6.96 ± 0.79	7.27 ± 0.99	7.31 ± 1.22	7.37 ± 1.20

Data are presented as mean ± standard deviation (SD), *n*, and %. BMI, body mass index.

**Table 2:** Blood glucose, lipid profile, and liver function parameters of participants in the control (CON), asparagus root extract supplementation (ARE), high-intensity interval training (HIIT), and combined HIIT with ARE supplementation (COM) groups at baseline and after the 12-week intervention.

Parameters	CON (*n* = 18)		ARE (*n* = 18)		HIIT (*n* = 18)		COM (*n* = 18)	
	Baseline	After	Baseline	After	Baseline	After	Baseline	After
BG (mg/dL)	89.94 ± 9.92	87.17 ± 10.08	102.33 ± 6.97	99.11 ± 4.78	89.33 ± 10.36	84.94 ± 10.90 *^,^**	86.72 ± 7.41	83.94 ± 6.71 *^,#^
TC (mg/dL)	202.50 ± 38.44	204.06 ± 32.44	197.61 ± 23.19	204.28 ± 28.38	202.61 ± 33.95	188.72 ± 37.25 *^,^**	189.94 ± 45.16	182.11 ± 37.75 *
TG (mg/dL)	111.00 ± 64.51	122.94 ± 68.63	109.66 ± 51.57	111.06 ± 51.16	119.83 ± 71.00	110.28 ± 47.43	94.50 ± 47.01	93.94 ± 43.70
HDLC (mg/dL)	55.27 ± 10.56	52.67 ± 9.58	53.61 ± 10.36	51.89 ± 9.04	51.83 ± 16.82	56.39 ± 15.91 *^,^**	55.44 ± 10.88	57.39 ± 10.30 *
LDLC (mg/dL)	135.88 ± 34.10	138.00 ± 29.34	131.77 ± 27.13	145.83 ± 30.31 *	138.77 ± 28.66	139.72 ± 35.39	122.50 ± 44.30	121.17 ± 40.18
VLDLC (mg/dL)	22.27 ± 12.92	24.50 ± 13.74	21.88 ± 10.35	22.22 ± 10.12	24.00 ± 14.18	23.33 ± 9.35	18.94 ± 9.40	20.39 ± 9.09
TC/HDLC ratio	3.75 ± 0.89	3.90 ± 0.89	3.85 ± 1.08	4.08 ± 1.05	4.19 ± 1.20	3.56 ± 1.13 *^,^**	3.67 ± 1.37	3.24 ± 0.73 *^,^**^,#^
LDLC/HDLC ratio	2.53 ± 0.79	2.72 ± 0.86	2.62 ± 1.07	2.95 ± 1.06 *	2.94 ± 1.11	2.72 ± 1.18	2.29 ± 0.90	2.17 ± 0.78
ALT (U/L)	-	-	19.47 ± 4.58	18.67 ± 12.12	-	-	16.36 ± 3.85	20.22 ± 11.27
AST (U/L)	-	-	23.83 ± 12.56	21.67 ± 4.98	-	-	21.61 ± 7.94	21.78 ± 5.63

Data are presented as mean ± SD. ALT, alanine aminotransferase; AST, aspartate aminotransferase; BG, blood glucose; HDLC, high-density lipoprotein cholesterol; LDLC, low-density lipoprotein cholesterol; TC, total cholesterol; TG, triglycerides; VLDLC, very-low-density lipoprotein cholesterol. * *p* < 0.05 vs. before intervention; ** *p* < 0.05 vs. CON group; ^#^ *p* < 0.05 vs. ARE group.

**Table 3:** White blood cell counts and percentages of participants in the control (CON), asparagus root extract supplementation (ARE), high-intensity interval training (HIIT), and combined HIIT with ARE supplementation (COM) groups at baseline and after the 12-week intervention.

Parameters	CON (*n* = 18)		ARE (*n* = 18)		HIIT (*n* = 18)		COM (*n* = 18)	
	Baseline	After	Baseline	After	Baseline	After	Baseline	After
Total white blood cell (×10^3^ cells/mm^3^)	7.64 ± 1.85	8.06 ± 1.58	7.43 ± 1.25	8.02 ± 1.27	7.48 ± 1.76	7.91 ± 1.59	7.23 ± 2.17	7.46 ± 2.01
Neutrophils (×10^3^ cells/mm^3^)	4.23 ± 1.48	4.39 ± 1.02	3.98 ± 0.72	4.39 ± 1.23	4.02 ± 1.10	3.93 ± 1.52	4.12 ± 1.47	4.25 ± 1.45
Neutrophils (%)	54.40 ± 7.66	54.47 ± 6.12	53.52 ± 4.90	54.08 ± 7.46	53.37 ± 5.60	52.14 ± 7.28	56.59 ± 8.37	56.33 ± 7.67
Eosinophils (cells/mm^3^)	177 ± 137	209 ± 160	255 ± 165	311 ± 226	199 ± 153	188 ± 91	163 ± 73	179 ± 81^#^
Eosinophils (%)	2.51 ± 1.95	2.66 ± 1.97	3.46 ± 2.34	3.95 ± 2.97	2.47 ± 1.39	2.33 ± 0.91 ^#^	2.28 ± 0.90	2.42 ± 1.02
Basophils (cells/mm^3^)	27 ± 14	30 ± 20	37 ± 14	34 ± 15	24 ± 8	28 ± 11	22 ± 18^#^	25 ± 17
Basophils (%)	0.38 ± 0.20	0.38 ± 0.24	0.49 ± 0.15	0.43 ± 0.19	0.33 ± 0.11	0.36 ± 0.17	0.31 ± 0.20	0.36 ± 0.23
Lymphocytes (×10^3^ cells/mm^3^)	2.72 ± 0.63	2.96 ± 0.84	2.73 ± 0.62	2.83 ± 0.51	2.75 ± 0.63	2.85 ± 0.95	2.52 ± 0.93	2.59 ± 0.81
Lymphocytes (%)	36.43 ± 6.76	36.53 ± 5.78	36.79 ± 5.57	35.79 ± 6.37	37.25 ± 5.65	39.01 ± 7.08	34.82 ± 7.35	35.01 ± 7.17
Monocytes (cells/mm^3^)	478 ± 148	481 ± 137	426 ± 105	454 ± 101	451 ± 157	486 ± 126	409 ± 91	415 ± 70
Monocytes (%)	6.25 ± 1.19	5.96 ± 1.13	5.72 ± 0.95	5.74 ± 1.41	6.55 ± 1.63	6.16 ± 1.01	5.97 ± 1.84	5.87 ± 1.53

Data are presented as mean ± SD. ^#^ *p* < 0.05 vs. ARE group.

## Data Availability

The data presented in this study are available on request from the corresponding author.

## Abbreviations

The following abbreviations are used in this manuscript:

20E	20-hydroxyecdysone
ALT	Alanine aminotransferase;
AMPK	AMP-activated protein kinase
ANCOVA	Analysis of covariance
ANOVA	Analysis of variance
ARE	Asparagus root extract
AST	Aspartate aminotransferase;
BG	Blood glucose
BMI	Body mass index
CAT	Catalase
COM	Combined intervention
CON	Control
DNPH	Dinitrophenylhydrazine
ELISA	Enzyme-linked immunosorbent assay
GC-MS	Gas chromatography-mass spectrometry
GLUT4	Glucose transporter 4
GPx	Glutathione peroxidase
GSH	Reduced glutathione
GSSG	Glutathione disulfide
HCL	Hydrochloric acid
HDLC	High-density lipoprotein cholesterol
HIIT	High-intensity interval training
HMG-CoA	3-hydroxy-3-methylglutaryl coenzyme A
HPLC	High-performance liquid chromatography
IL	Interleukin
LDLC	Low-density lipoprotein cholesterol
M2	Alternatively activated macrophages
MARK	Mitogen-activated protein kinase
MDA	Malondialdehyde
NADPH	Nicotinamide adenine dinucleotide phosphate
NF-κB	Nuclear factor- kappa B
Nrf2	Nuclear factor erythroid 2-related factor 2
PC	Protein carbonyl
PGC-1α	Peroxisome proliferator-activated receptor γ coactivator 1α
PPAR-α	Peroxisome proliferator-activated receptor alpha
ROS	Reactive oxygen species
SD	Standard deviation
SOD	Superoxide dismutase
TBAR	Thiobarbituric acid reactive substance
TC	Total cholesterol
TCA	Trichloroacetic acid
TG	Triglyceride
Th2	T helper 2
TNF-α	Tumor-necrosis factor-alpha
VLDLC	Very-low-density lipoprotein cholesterol
WBC	White blood cell

## References

[1] Ahmed S.K., Mohammed R.A. (2025). Obesity: Prevalence, causes, consequences, management, preventive strategies and future research directions. Metabol. Open.

[2] Powell-Wiley T.M., Poirier P., Burke L.E., Després J.P., Gordon-Larsen P., Lavie C.J., Lear S.A., Ndumele C.E., Neeland I.J., Sanders P. (2021). Obesity and Cardiovascular Disease: A Scientific Statement From the American Heart Association. Circulation.

[3] Manna P., Jain S.K. (2015). Obesity, Oxidative Stress, Adipose Tissue Dysfunction, and the Associated Health Risks: Causes and Therapeutic Strategies. Metab. Syndr. Relat. Disord..

[4] Jankovic A., Korac A., Buzadzic B., Otasevic V., Stancic A., Daiber A., Korac B. (2015). Redox implications in adipose tissue (dys)function--A new look at old acquaintances. Redox Biol..

[5] Asatiani N., Sapojnikova N., Kartvelishvili T., Asanishvili L., Sichinava N., Chikovani Z. (2025). Blood Catalase, Superoxide Dismutase, and Glutathione Peroxidase Activities in Alcohol- and Opioid-Addicted Patients. Medicina.

[6] Shi C., Zhu L., Chen X., Gu N., Chen L., Zhu L., Yang L., Pang L., Guo X., Ji C. (2014). IL-6 and TNF-α induced obesity-related inflammatory response through transcriptional regulation of miR-146b. J. Interferon Cytokine Res..

[7] Atakan M.M., Li Y., Koşar Ş.N., Turnagöl H.H., Yan X. (2021). Evidence-Based Effects of High-Intensity Interval Training on Exercise Capacity and Health: A Review with Historical Perspective. Int. J. Environ. Res. Public Health.

[8] Reljic D. (2025). High-Intensity Interval Training as Redox Medicine: Targeting Oxidative Stress and Antioxidant Adaptations in Cardiometabolic Disease Cohorts. Antioxidants.

[9] Ouerghi N., Fradj M.K.B., Duclos M., Bouassida A., Feki M., Weiss K., Knechtle B. (2022). Effects of High-Intensity Interval Training on Selected Adipokines and Cardiometabolic Risk Markers in Normal-Weight and Overweight/Obese Young Males-A Pre-Post Test Trial. Biology.

[10] Wang S., Zhou H., Zhao C., He H. (2022). Effect of Exercise Training on Body Composition and Inflammatory Cytokine Levels in Overweight and Obese Individuals: A Systematic Review and Network Meta-Analysis. Front. Immunol..

[11] Pegiou E., Mumm R., Acharya P., de Vos R.C.H., Hall R.D. (2019). Green and White *Asparagus* (*Asparagus officinalis*): A Source of Developmental, Chemical and Urinary Intrigue. Metabolites.

[12] Denben B., Sripinyowanich S., Ruangthai R., Phoemsapthawee J. (2023). Beneficial Effects of *Asparagus officinalis* Extract Supplementation on Muscle Mass and Strength following Resistance Training and Detraining in Healthy Males. Sports.

[13] Cai Y.J., Dai J.Q., Fang J.G., Ma L.P., Hou L.F., Yang L., Liu Z.L. (2002). Antioxidative and free radical scavenging effects of ecdysteroids from *Serratula strangulata*. Can. J. Physiol. Pharmacol..

[14] Bhardwaj M., Mamadalieva N.Z., Chauhan A.K., Kang S.C. (2019). α-Ecdysone suppresses inflammatory responses via the Nrf2 pathway in lipopolysaccharide-stimulated RAW 264.7 cells. Int. Immunopharmacol..

[15] Catalán R.E., Martinez A.M., Aragones M.D., Miguel B.G., Robles A., Godoy J.E. (1985). Alterations in rat lipid metabolism following ecdysterone treatment. Comp. Biochem. Physiol. B.

[16] Gibala M.J., Little J.P., Macdonald M.J., Hawley J.A. (2012). Physiological adaptations to low-volume, high-intensity interval training in health and disease. J. Physiol..

[17] Little J.P., Gillen J.B., Percival M.E., Safdar A., Tarnopolsky M.A., Punthakee Z., Jung M.E., Gibala M.J. (2011). Low-volume high-intensity interval training reduces hyperglycemia and increases muscle mitochondrial capacity in patients with type 2 diabetes. J. Appl. Physiol..

[18] Hood M.S., Little J.P., Tarnopolsky M.A., Myslik F., Gibala M.J. (2011). Low-volume interval training improves muscle oxidative capacity in sedentary adults. Med. Sci. Sports Exerc..

[19] Dolati S., Namiranian K., Amerian R., Mansouri S., Arshadi S., Azarbayjani M.A. (2020). The Effect of Curcumin Supplementation and Aerobic Training on Anthropometric Indices, Serum Lipid Profiles, C-Reactive Protein and Insulin Resistance in Overweight Women: A Randomized, Double-Blind, Placebo-Controlled Trial. J. Obes. Metab. Syndr..

[20] Yang G., Yang W., Kiarasi F. (2025). Polyphenol-Based Nutritional Strategies Combined with Exercise for Brain Function and Glioma Control: Focus on Epigenetic Modifications, Cognitive Function, Learning and Memory Processes. Food Sci. Nutr..

[21] Mongraykang J., Padkao T., Boonla O., Teethaisong Y., Roengrit T., Koowattanatianchai S., Prasertsri P. (2025). Effects of Asparagus Powder Supplementation on Glycemic Control, Lipid Profile, and Oxidative Stress in Overweight and Obese Adults: An Exploratory Randomized Controlled Trial. Life.

[22] Tjønna A.E., Lee S.J., Rognmo Ø., Stølen T.O., Bye A., Haram P.M., Loennechen J.P., Al-Share Q.Y., Skogvoll E., Slørdahl S.A. (2008). Aerobic interval training versus continuous moderate exercise as a treatment for the metabolic syndrome: A pilot study. Circulation.

[23] Kessler H.S., Sisson S.B., Short K.R. (2012). The potential for high-intensity interval training to reduce cardiometabolic disease risk. Sports Med..

[24] Musa D.I., Adeniran S.A., Dikko A.U., Sayers S.P. (2009). The effect of a high-intensity interval training program on high-density lipoprotein cholesterol in young men. J. Strength Cond. Res..

[25] Olas B. (2024). A Review of the Pro-Health Activity of *Asparagus officinalis* L. and Its Components. Foods.

[26] Hernández-Lepe M.A., Olivas-Aguirre F.J., Gómez-Miranda L.M., Hernández-Torres R.P., Manríquez-Torres J.J., Ramos-Jiménez A. (2019). Systematic Physical Exercise and Spirulina maxima Supplementation Improve Body Composition, Cardiorespiratory Fitness, and Blood Lipid Profile: Correlations of a Randomized Double-Blind Controlled Trial. Antioxidants.

[27] Nemati S., Tadibi V., Hoseini R. (2021). How combined aerobic training and pomegranate juice intake affect lipid profile? A clinical trial in men with type 2 diabetes. Biomed. Human Kinet..

[28] Fisher-Wellman K., Bloomer R.J. (2009). Acute exercise and oxidative stress: A 30 year history. Dyn. Med..

[29] Avloniti A., Chatzinikolaou A., Deli C.K., Vlachopoulos D., Gracia-Marco L., Leontsini D., Draganidis D., Jamurtas A.Z., Mastorakos G., Fatouros I.G. (2017). Exercise-Induced Oxidative Stress Responses in the Pediatric Population. Antioxidants.

[30] Souza-Silva A.A., Moreira E., de Melo-Marins D., Schöler C.M., de Bittencourt P.I., Laitano O. (2016). High intensity interval training in the heat enhances exercise-induced lipid peroxidation, but prevents protein oxidation in physically active men. Temperature.

[31] Samjoo I.A., Safdar A., Hamadeh M.J., Raha S., Tarnopolsky M.A. (2013). The effect of endurance exercise on both skeletal muscle and systemic oxidative stress in previously sedentary obese men. Nutr. Diabetes.

[32] Petersen A.M., Pedersen B.K. (2005). The anti-inflammatory effect of exercise. J Appl Physiol..

[33] Gleeson M., Bishop N.C., Stensel D.J., Lindley M.R., Mastana S.S., Nimmo M.A. (2011). The anti-inflammatory effects of exercise: Mechanisms and implications for the prevention and treatment of disease. Nat. Rev. Immunol..

[34] Aggarwal D., Chaudhary M., Mandotra S.K., Tuli H.S., Chauhan R., Joshi N.C., Kaur D., Dufossé L., Chauhan A. (2025). Anti-inflammatory potential of quercetin: From chemistry and mechanistic insight to nanoformulations. Curr. Res. Pharmacol. Drug Discov..

[35] García-Mediavilla V., Crespo I., Collado P.S., Esteller A., Sánchez-Campos S., Tuñón M.J., González-Gallego J. (2007). The anti-inflammatory flavones quercetin and kaempferol cause inhibition of inducible nitric oxide synthase, cyclooxygenase-2 and reactive C-protein, and down-regulation of the nuclear factor kappaB pathway in Chang Liver cells. Eur. J. Pharmacol..

[36] Wijesekara T., Luo J., Xu B. (2024). Critical review on anti-inflammation effects of saponins and their molecular mechanisms. Phytother. Res..

[37] Ghanim H., Sia C.L., Abuaysheh S., Korzeniewski K., Patnaik P., Marumganti A., Chaudhuri A., Dandona P. (2010). An antiinflammatory and reactive oxygen species suppressive effects of an extract of *Polygonum cuspidatum* containing resveratrol. J. Clin. Endocrinol. Metab..

[38] Qiu L., Gao C., Wang H., Ren Y., Li J., Li M., Du X., Li W., Zhang J. (2023). Effects of dietary polyphenol curcumin supplementation on metabolic, inflammatory, and oxidative stress indices in patients with metabolic syndrome: A systematic review and meta-analysis of randomized controlled trials. Front. Endocrinol..

[39] Haghighatdoost F., Gholami A., Hariri M. (2020). Effect of grape polyphenols on selected inflammatory mediators: A systematic review and meta-analysis randomized clinical trials. Excli J..

[40] Radak Z., Chung H.Y., Koltai E., Taylor A.W., Goto S. (2008). Exercise, oxidative stress and hormesis. Ageing Res. Rev..

[41] Pedersen B.K. (2019). The Physiology of Optimizing Health with a Focus on Exercise as Medicine. Annu. Rev. Physiol..

[42] Shirato K., Koda T., Takanari J., Sakurai T., Ogasawara J., Imaizumi K., Ohno H., Kizaki T. (2018). Anti-Inflammatory Effect of ETAS^®^50 by Inhibiting Nuclear Factor-κB p65 Nuclear Import in Ultraviolet-B-Irradiated Normal Human Dermal Fibroblasts. Evid. Based Complement. Alternat. Med..

[43] Sobhy Y., Mahgoub S., Abo-Zeid Y., Mina S.A., Mady M.S. (2024). In-Vitro Cytotoxic and Anti-Inflammatory Potential of Asparagus Densiflorus Meyeri and its Phytochemical Investigation. Chem. Biodivers..

[44] Soltani N., Marandi S.M., Kazemi M., Esmaeil N. (2020). Combined All-Extremity High-Intensity Interval Training Regulates Immunometabolic Responses through Toll-Like Receptor 4 Adaptors and A20 Downregulation in Obese Young Females. Obes. Facts.

[45] Pérez-Castillo I.M., Rueda R., Bouzamondo H., Aparicio-Pascual D., Valiño-Marques A., López-Chicharro J., Segura-Ortiz F. (2025). Does Lifelong Exercise Counteract Low-Grade Inflammation Associated with Aging? A Systematic Review and Meta-Analysis. Sports Med..

[46] Almuraikhy S., Sellami M., Al-Amri H.S., Domling A., Althani A.A., Elrayess M.A. (2023). Impact of Moderate Physical Activity on Inflammatory Markers and Telomere Length in Sedentary and Moderately Active Individuals with Varied Insulin Sensitivity. J. Inflamm. Res..

[47] Wu D., Molofsky A.B., Liang H.E., Ricardo-Gonzalez R.R., Jouihan H.A., Bando J.K., Chawla A., Locksley R.M. (2011). Eosinophils sustain adipose alternatively activated macrophages associated with glucose homeostasis. Science.

[48] Qiu Y., Nguyen K.D., Odegaard J.I., Cui X., Tian X., Locksley R.M., Palmiter R.D., Chawla A. (2014). Eosinophils and type 2 cytokine signaling in macrophages orchestrate development of functional beige fat. Cell.

[49] Walsh N.P., Gleeson M., Shephard R.J., Gleeson M., Woods J.A., Bishop N.C., Fleshner M., Green C., Pedersen B.K., Hoffman-Goetz L. (2011). Position statement. Part one: Immune function and exercise. Exerc. Immunol. Rev..

[50] Scott H.A., Wood L.G., Williams E.J., Weaver N., Upham J.W. (2022). Comparing the Effect of Acute Moderate and Vigorous Exercise on Inflammation in Adults with Asthma: A Randomized Controlled Trial. Ann. Am. Thorac. Soc..

[51] Moraes-Ferreira R., Brandao-Rangel M.A.R., Gibson-Alves T.G., Silva-Reis A., Souza-Palmeira V.H., Aquino-Santos H.C., Frison C.R., Oliveira L.V.F., Albertini R., Vieira R.P. (2022). Physical Training Reduces Chronic Airway Inflammation and Mediators of Remodeling in Asthma. Oxid. Med. Cell Longev..

[52] Viriyautsahakul V., Soontornmanokul T., Komolmit P., Jiamjarasrangsi W., Treeprasertsuk S. (2013). What is the normal serum alanine aminotransferase (ALT) value for Thai subjects with the low risk of liver diseases?. Chulalongkorn Med. J..

[53] Varra F.N., Varras M., Varra V.K., Theodosis-Nobelos P. (2025). Mechanisms Linking Obesity with Non-Alcoholic Fatty Liver Disease (NAFLD) and Cardiovascular Diseases (CVDs)-The Role of Oxidative Stress. Curr. Issues Mol. Biol..

[54] Dao M.C., Subar A.F., Warthon-Medina M., Cade J.E., Burrows T., Golley R.K., Forouhi N.G., Pearce M., Holmes B.A. (2019). Dietary assessment toolkits: An overview. Public Health Nutr..

[55] Baecke J.A., Burema J., Frijters J.E. (1982). A short questionnaire for the measurement of habitual physical activity in epidemiological studies. Am. J. Clin. Nutr..

[56] Jalayondeja C., Jalayondeja W., Vachalathiti R., Bovonsunthonchai S., Sakulsriprasert P., Kaewkhuntee W., Bunprajun T., Upiriyasakul R. (2015). Cross-Cultural Adaptation of the Compendium of Physical Activity: Thai Translation and Content Validity. J. Med. Assoc. Thai.

[57] Dinan L., Dioh W., Veillet S., Lafont R. (2021). 20-Hydroxyecdysone, from Plant Extracts to Clinical Use: Therapeutic Potential for the Treatment of Neuromuscular, Cardio-Metabolic and Respiratory Diseases. Biomedicines.

[58] Padkao T., Prasertsri P. (2025). The Impact of Modified Tabata Training on Segmental Fat Accumulation, Muscle Mass, Muscle Thickness, and Physical and Cardiorespiratory Fitness in Overweight and Obese Participants: A Randomized Control Trial. Sports.

[59] Padkao T., Prasertsri P. (2025). Effects of High-Intensity Intermittent Training Combined with *Asparagus officinalis* Extract Supplementation on Cardiovascular and Pulmonary Function Parameters in Obese and Overweight Individuals: A Randomized Control Trial. J. Funct. Morphol. Kinesiol..

[60] Kukongviriyapan U., Kukongviriyapan V., Pannangpetch P., Donpunha W., Sripui J., Sae-Eaw A., Boonla O. (2015). Mamao Pomace Extract Alleviates Hypertension and Oxidative Stress in Nitric Oxide Deficient Rats. Nutrients.

[61] Intakhiao S., Prakobkaew N., Buddhisa S., Boonla O., Teethaisong Y., Koowattanatianchai S., Prasertsri P. (2025). Ameliorative Effects of Triphala Supplementation on Oxidative Stress and Inflammation in Individuals with Post-COVID-19 Condition: A Preliminary Randomized Controlled Trial. Glob. Adv. Integr. Med. Health.

[62] Burnett R.W., D’Orazio P., Fogh-Andersen N., Kuwa K., Külpmann W.R., Larsson L., Lewnstam A., Maas A.H., Mager G., Spichiger-Keller U. (2001). IFCC recommendation on reporting results for blood glucose. Clin. Chim. Acta.

